# Taste disorder in facial onset sensory and motor neuronopathy: a case report

**DOI:** 10.1186/s12883-020-01639-x

**Published:** 2020-02-29

**Authors:** Nobuhiko Ohashi, Jin Nonami, Minori Kodaira, Kunihiro Yoshida, Yoshiki Sekijima

**Affiliations:** 1 Department of Medicine (Neurology and Rheumatology), Shinshu University School of Medicine, Matsumoto, Japan; 2 Division of Neurogenetics, Department of Brain Disease Research, Shinshu University School of Medicine, 3-1-1 Asahi, Matsumoto, 390-8621 Japan; 3 Institute for Biomedical Sciences, Shinshu University, Matsumoto, Japan

**Keywords:** Facial onset sensory and motor neuronopathy, Taste disorder, Solitary nucleus

## Abstract

**Background:**

Taste disorder is a common symptom in the general population. Several studies have shown that patients with neurological disorders, such as amyotrophic lateral sclerosis and Parkinson’s disease, develop taste disturbance. Facial onset sensory and motor neuronopathy (FOSMN) is a rare disease characterized by sensory disturbance and weakness spreading from the face to the limbs caudally. We describe a patient with FOSMN who showed taste disorder as the sole initial symptom.

**Case presentation:**

A 49-year-old man who smoked cigarettes developed taste disturbance. Despite using zinc supplements, an herbal medication, and an ointment, his taste disorder worsened. 4 years later, a tingling feeling emerged at the tip of his tongue and gradually spread to his entire lips. At 55 years of age, he showed difficulty in swallowing, followed by facial paresthesia, muscle atrophy, and weakness in the face and upper limbs without apparent upper motor neuron sign. Cessation of smoking did not improve his taste disturbance, and he was unable to discriminate different tastes on the entire tongue. In an electrogustometric study, electrical stimulation did not induce any type of taste sensation. Blink reflex showed delayed or diminished R2 responses. Needle electromyography revealed severe chronic neurogenic changes in the tongue and masseter muscles. Mild chronic neurogenic changes were also observed in the limbs. In the thoracic paraspinal muscles, active neurogenic changes were detected. Findings of hematological and cerebrospinal fluid analyses, and magnetic resonance images of the brain and spinal cord were unremarkable. One cycle of intravenous immunoglobulin therapy did not improve his symptoms. We diagnosed him as having FOSMN with the sole initial symptom of taste disorder. Nine years after the onset of taste disorder, he developed impaired sensation of touch in the right upper limb and required tube feeding and ventilator support.

**Conclusion:**

Taste disorder can be the initial manifestation of FOSMN and might involve the solitary nucleus.

## Background

Taste disorder is among the common symptoms encountered in routine medical practice. Indeed, previous studies have reported that 0.6–20% of the general population suffer from taste dysfunction [1–5]. The taste sensation can be disturbed by various factors and disorders, such as aging, drug, zinc deficiency, infection, and head surgery [6]. Furthermore, patients with neurological disorders, such as amyotrophic lateral sclerosis (ALS), seizures, and Alzheimer’s and Parkinson’s diseases, may present with taste disturbance [7–9].

Facial onset sensory and motor neuronopathy (FOSMN) is a rare disease characterized by sensory disturbance and weakness of the face followed by muscle weakness in the limbs [10–12]. FOSMN might be a variant of ALS resulting from an irreversible disease progression with a lack of response to immunotherapies as well as deposition of TAR DNA-binding protein 43 (TDP-43) in the nervous systems [13–15]. However, the clinical pictures and pathophysiology of FOSMN have not been well established. Herein, we report a patient with FOSMN who showed taste disorder as the sole initial symptom of the disease.

## Case presentation

**Fig. 1:**
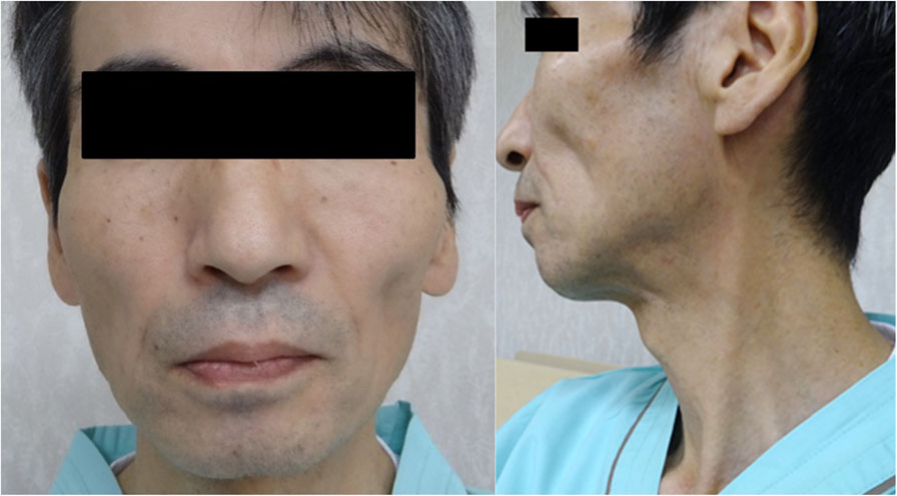
Atrophy of the temporal, masseter, and sternocleidomastoid muscles

**Fig. 2:**
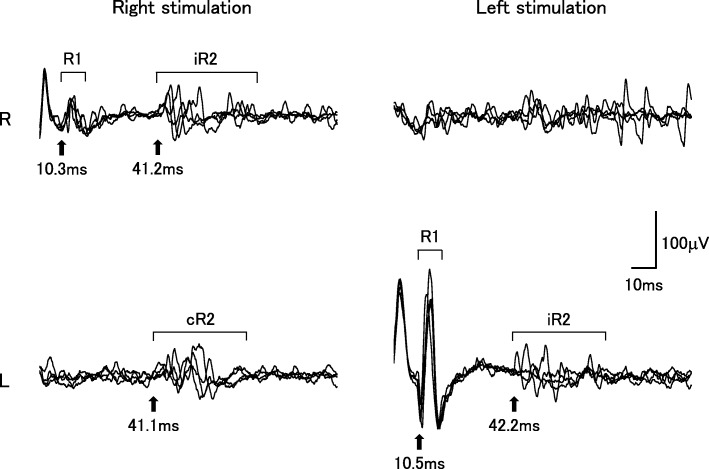
Evaluation of the blink reflex. The blink reflex shows impaired brainstem function with delayed or absent R2 responses after ipsilateral and contralateral stimulation. Arrows indicate latency of each response. Normal values (ms): R1 10.5 ± 0.8, ipsilateral R2 (iR2) 30.5 ± 3.4, and contralateral R2 (cR2) 30.5 ± 4.4 [16]

The patient was a Japanese man without a significant family history. He was a smoker and had noticed impairment of his sense of smell following a sinusitis surgery at the age of 20 years. He had no history of medication use. At the age of 49 years, he developed taste disturbance with a lingering salty taste in the mouth and tasted barley tea as salty noodle soup. One year later, he visited a local otolaryngology clinic. He was suspected to have zinc deficiency, and oral zinc supplements were administered. However, his taste disturbance progressed to a sweet taste in addition to the salty taste within the month. At the age of 53 years, a tingling feeling emerged at the tip of the tongue and gradually spread to his entire lips. Despite using an herbal medication and an ointment, these abnormal sensations remained. At the age of 55 years, he showed mild difficulty in swallowing. During the following year, he stopped smoking, but it did not improve his taste disturbance. Subsequently, he developed speech difficulties and muscle wasting of the neck and upper limbs and was referred to our hospital. He presented with mild impairment of the smell sensation. Neither ptosis nor an eye movement disorder was apparent. He showed numbness around the mouth. Touch and pinprick sensations were decreased in the area of the third division of the bilateral trigeminal nerves. Moderate muscle weakness was detected in the orbicularis oculi and oris muscles. Muscle atrophy was noted in the temporal, masseter, and sternocleidomastoid muscles (Fig. 1). He had mild dysarthria with remarkable atrophy and fasciculation of the tongue. Corneal and gag reflexes were absent. Severe weakness of the neck extensors caused his head to drop. Further, mild muscle weakness was detected in the left upper limb. Sensory disturbances in the trunk and limbs were not apparent. Bilateral tendon reflexes were normal in the upper limbs and slightly brisk in the lower limbs without pathological reflexes. Neither cerebellar ataxia nor dysautonomia was apparent. Hematological analysis revealed mild elevation of creatinine kinase (392 U/l). Results of complete blood count; electrolytes, including sodium and zinc; vitamin B1 and B12; total cholesterol and triglyceride; blood sugar and HbA1c; liver and renal functions; autoantibodies including anti-SS-A and SS-B, anti-GM1, and GQ1b IgG; and C-reactive protein were normal. Cerebrospinal fluid analysis revealed unremarkable findings. Magnetic resonance imaging (MRI) of the brain was unremarkable. MRI of the spinal cord showed mild cervical spondylosis without spinal cord atrophy. Pulmonary function test showed mild decrement of % vital capacity (76.4%). Results of a nerve conduction study were normal except for reduced F-wave occurrence in the median nerve (30%). In needle electromyography, chronic neurogenic changes with severely decreased number of motor units and high amplitude/long duration motor unit potentials were apparent in the tongue and masseter muscles. Mild chronic neurogenic changes were also observed in the biceps brachii, first dorsal interosseous, quadriceps, and gastrocnemius medialis muscles. Active neurogenic changes, including fasciculation potentials were unapparent in these muscles. In the thoracic paraspinal muscles, fibrillation potentials and positive sharp waves were detected. Regarding the blink reflex, both sides of the R2 components after ipsilateral stimulation were delayed, while the right side of the R2 component after left stimulation was absent (Fig. 2). An assessment using the disc test revealed that the patient was unable to discriminate different tastes in the entire tongue. In an electrogustometric study, electrical stimulation of all segments of the tongue failed to induce any type of taste sensation. Laryngoscopy showed no evidence of inflammation and/or masses within the larynx and pharynx. One session of intravenous immunoglobulin (IVIG) therapy (2 g/kg body weight over 5 days) did not improve any of his symptoms and signs. Based on these results, we diagnosed him as having FOSMN with the sole initial symptom of taste disorder. Nine years after the onset of the condition, he developed impaired sensation of touch in the right upper limb and required mechanical ventilation and nutritional support via tube feeding. Subsequent electromyography studies were not performed.

## Discussion

FOSMN is characterized by an initial manifestation of somatosensory sensory disturbance and/or weakness in the face; however, our patient developed taste disorder as the initial symptom. Smoking may have facilitated the onset and progression of taste disorder in our patient, but subjects with chronic exposure to cigarette smoke can experience improved taste disturbance soon after the cessation of smoking [17]. Thus, we think that taste disorder in our patient was associated with FOSMN.

Approximately 50 patients with FOSMN have been described in the literature to date [10, 11, 18], with 8 of them showing taste disturbance (Table 1). Most patients with taste disorder developed taste disturbance during the course of the disease. However, Vucic et al. described a 42-year-old man (patient 1 in Table 1) who developed taste disturbance with perioral sensory disturbance as an initial symptom. He was positive for anti-sulfatide antibody; however, he did not respond to immunotherapies, including prednisone, azathioprine, and mycophenolate mofetil. Contrastingly, our patient developed taste disturbance as the sole initial symptom with other manifestations developing more than 4 years after the onset of taste disorder. Although our patient did not respond to IVIG, he was alive without tube feeding or mechanical ventilation for over 8 years, which is similar to the case of a patient in a report by Vucic et al. [10]. Except for taste disturbance, the clinical characteristics of FOSMN with taste disorder were not markedly different from those without taste disorder.

**Table 1:** Clinical features in FOSMN patients with a taste disorder

Patient	Age at onset, y/sex	Initial symptoms	Clinical course	Autoantibodies	Response to treatments	Reference
Present case	49/M	Taste disorder	Weakness of the upper limbs at age of 55 Mechanical ventilation and tube feeding at age of 58	(−)	No response to IVIG	
1	42/M	Taste disorder Perioral paresthesia and numbness	Weakness of the neck and shoulder girdles at the age of 50 Tube feeding at the age of 51 Died at the age of 52 due to pulmonary embolism	(+), Anti-sulfatide antibody	No response to PDN, AZA and MMF	[10]
2	63/F	Facial paresthesia	NA	(−)	A part of patients 2–8 were treated with IVIG, but none of them responded to the treatment.	[18]
3	44/F	Facial paresthesia	NA	(−)		[18]
4	40/M	Dysarthria	NA	(−)		[18]
5	41/M	Facial paresthesia and weakness	NA	(−)		[18]
6	62/F	Facial paresthesia and weakness	NA	(−)		[18]
7	50/F	Facial and upper limbs’ paresthesia and weakness	NA	(−)		[18]
8	51/M	Facial paresthesia and weakness Weakness of upper limbs	NA	(−)		[18]

*IVIG* intravenous immunoglobulin, *PDN* prednisone, *AZA* azathioprine, *MMF* mycophenolate mofetil

Similar to patients with FOSMN who have taste disorder, a recent study reported changes in taste perception in patients with ALS who have dysphagia; among 32 patients, 8 of 11 (73%) with an enteral tube and 5 of 21 (29%) without an enteral tube complained of taste disturbance [9]. This result indicates that many patients with ALS may develop taste abnormalities. However, to our knowledge, there have been no reports of patients with ALS developing taste disorder as the initial manifestation of the disease.

In patients with neurodegenerative diseases, a taste disorder might be due to dysfunction of the gustatory tract, which consists of the facial nerve in the anterior two-thirds and the glossopharyngeal nerve in the posterior third of the tongue, the nucleus of the solitary tract, the thalamus, and the primary gustatory cortex. Brainstem pathologies seem to differ between patients with FOSMN and those with ALS. Autopsy studies examining patients with FOSMN have revealed neuronal loss and reactive astrocytosis with TDP-43 inclusions in the motor and sensory nuclei of the trigeminal and facial nerves, dorsal motor nucleus of the vagal nerve, nucleus ambiguous, nucleus of the solitary tract, and hypoglossal nerve [13–15]. On the other hand, TDP-43 pathologic involvement of cortical and subcortical white matter is rare [13, 15]. Neuronal loss and gliosis are not apparent in the thalamus, although deposition of TDP-43 can be detected similarly to sensory and motor nuclei in the brainstem [15]. These findings indicate that taste disturbance in patients with FOSMN might result from impairment of the solitary nucleus. Contrastingly, the brainstem pathology of ALS is characterized by neuronal loss and reactive astrocytosis with TDP-43 pathology in the motor nuclei of the trigeminal and facial nerves, dorsal motor nucleus of the vagal nerve, and nucleus of the hypoglossal nerve. TDP-43 deposition in the solitary nucleus is rare in ALS [19], and loss of the solitary nucleus was reported in only one Japanese ALS patient with *fused in sarcoma* gene mutation [20]. Given the rare involvement of the solitary nucleus in ALS, brain degeneration in FOSMN probably involves a different mechanism.

## Conclusions

For the first time in the literature, we describe a patient with FOSMN who developed taste disturbance as the sole initial manifestation of the disease. In addition to somatosensory and/or motor dysfunction, taste disturbance can be an initial symptom in patients with FOSMN.

## Data Availability

All data generated or analyzed during this study are included in this published article.

## Abbreviations

ALS: amyotrophic lateral sclerosis
FOSMN: facial onset sensory and motor neuronopathy
IVIG: intravenous immunoglobulin
MRI: magnetic resonance imaging
TDP-43: TAR DNA-binding protein 43
